# Beyond mere respect: new perspectives on dignity for healthcare workplace learning

**DOI:** 10.3389/fmed.2023.1274364

**Published:** 2024-01-16

**Authors:** Christiane Klinner, Amabile Borges Dario, Amani Bell, Gillian Nisbet, Merrolee Penman, Lynn V. Monrouxe

**Affiliations:** Faculty of Medicine and Health, The University of Sydney, Sydney, NSW, Australia

**Keywords:** professional dignity, healthcare professions education, work-integrated learning, workplace learning, qualitative research, allied health, health sciences students, clinical educators

## Abstract

**Introduction:**

Although dignity in workplace learning in healthcare is gathering interest, we know little about stakeholders’ conceptualizations in this area across professional groups. Dignity breaches in workplace learning are common, often with serious and long-lasting consequences for the affected. Conceptualizations shape behaviours and experiences. To prevent dignity violations in students’ learning, it is thus important to understand stakeholders’ understandings of the topic. This study therefore explores the dignity conceptualizations around workplace learning that students, placement educators and university staff hold across seven allied health professional groups.

**Methods:**

Using a social constructionist perspective, we conducted individual and group narrative interviews (*n* = 51) with students, placement educators and university workplace learning staff from seven allied health professional groups. We used the 5-step Framework Analysis to explore and develop themes, identifying differences and similarities across stakeholder groups.

**Results:**

We identified eight distinct, yet interrelated, dimensions of dignity from participants’ narratives: dignity as respect, dignity as self-x (the various relationships we have with ourselves), dignity as feeling safe, dignity as understanding otherness, dignity as supporting others, dignity as equality, dignity as professionalism, and dignity as belonging. Dignity as respect was identified across all participants, although mutual respect and a culture of respect were only present in academic participants’ talk. The remaining seven dimensions all present important factors extending our understanding of the construct of dignity.

**Discussion:**

In line with existing research, our study identifies the absence of an unambiguous, positive conceptualization of dignity in workplace learning among stakeholders. It adds novelty in two ways: by identifying dignity dimensions that require informed action beyond respecting others, and by revealing a tension between dignity as professionalism and dignity as equality. We suggest revising existing dignity concepts in workplace learning to address this tension and to reinforce that active care, team integration and skilled support are all non-negotiable elements of dignified behaviour within workplace learning.

## Introduction

The construct of dignity in the healthcare workplace is deemed core to good practice, being highlighted across numerous healthcare policies internationally, e.g., (1, 2). However, dignity is often confounded with *respect.* But it is not the same. Indeed, research has identified that the construct of dignity changes according to where and who is asked, e.g., (3–9). As such dignity is a sociocultural concept with antecedents, attributes and consequences (9). For example, achieving dignity in nursing is associated with empowerment for the persons involved, including enhancement of positive coping, well-being, self-esteem, integrity, hope, and control (9, 10). As a complex abstract construct, and in line with a social constructionist worldview, it is subject to individuals’ contextual understanding being affected by “*environmental and structural factors and organizational relationships*” [(10), p. 2]. Accordingly, it has been recommended that researchers investigate the meaning of dignity in terms of differing contexts and population groups. This is especially important so we can develop nuanced understandings of how we might uphold dignity in the healthcare workplace, as dignity breaches can have serious negative consequences personally and organizationally (11). In this article we explore a range of stakeholders’ explicit understandings of what dignity *in workplace learning* means to them, discuss the relationships and tensions between these dimensions, and propose how dignity can be usefully delineated from the concept of respect. Ultimately, we aim to make dignity a more accessible and useful concept for all stakeholders involved in healthcare workplace learning encounters.

### Conceptualizing dignity

The concept of dignity has been extensively discussed and debated by scholars throughout centuries. In its basic form it has been described as “*being worthy of being appreciatively acknowledged as worthy*” [(12), p. 253]. Put differently, dignity means living in accordance with one’s standards and values and respecting others’ standards and values (13). Dignity thus includes both an outward and an inward looking perspective (14): that is, an “*ability to establish a sense of self-worth and self-respect, and to appreciate the respect of others*” (15). It also encapsulates a *right to* and a *responsibility for* dignified behavior, calling for both self-assertion and self-renunciation (12). In this way, dignity comprises a highly relational, context-dependent, and dynamic concept which is created, upheld or violated, as a result of self-image, social interaction and moral behaviors (10, 16, 17). It relates to every person and to all areas of social interaction. In sum, it has been argued that dignity can be associated with “*what we do*… *what we suffer*…and sometimes to *what we are*” [(18), p. 202]. Of course, these elements are interrelated. Historically, dignity has been associated as being a property of a human being: “the essential and unavoidable core of our *humanity*,” [(19), p. 17] and this view has persisted, being embedded in societal values and laws. Following this supposition, it is logical that if we all have dignity, then we cannot treat people in any way that we please. We ought to treat others with the dignity they deserve.

This moral imperative is typically understood by banning two key types of behaviors: instrumentalization and degrading treatment/humiliation of others (18). And while the degrading treatment and humiliation of others are obvious behaviors to avoid (and we discuss this further in our section on dignity breaches during workplace learning), instrumentalization is less often discussed. Instrumentalization is linked to a lack of respect for the other and includes acts such as removing a person’s autonomy, imposing our own goals on them, taking away their intrinsic value (18).

### Conceptualising dignity in the workplace

The subjective elements of dignity at work (for example self-esteem, autonomy and meaningful work) have been the focus of many studies (20). Indeed, this aspect has recently focused on dignity breaches by peers and those in authority such as bullying and harassment (i.e., the degrading treatment and humiliation of others in the workplace), leading to dignity at work policies being developed across the world. Indeed, such dignity at work legislations commonly focus on the provision of safe working environments recognizing employees’ rights to be treated with dignity and respect, free from bullying, harassment and sexual harassment (as such, tend to omit actions around instrumentalization as discussed above) (21, 22). However, this aspect of dignity is not forgotten. The construct of ‘dignity in labor’ focuses on the central role that work plays in human dignity, including: “the right to decent work” with opportunities for people to have access to safe working conditions with well-paid jobs and secure working contracts (20). Here the focus is on “the dignity of the process of organization” [(23), p. 2]. In other words, increasing our focus towards the dignity *of* work, as well as dignity *at* work (20, 23, 24).

### Conceptualisations of dignity during healthcare workplace learning

In the context of healthcare learning, stakeholders comprise university staff, healthcare professionals, students, patients and their families: interacting in the often-overlapping areas of dignity at work, dignity while learning, and dignity while accessing professional healthcare (20, 23, 25, 26). While dignity at work and, more specifically, dignity in healthcare work, especially in nursing, has been extensively discussed (13, 16, 17, 25, 27–35), we know little about dignity in workplace *learning* in healthcare. However, due to growing concerns around professionalism breaches during workplace learning, and in particular around bullying and harassment [discussed further below (26, 36–45)], this topic is gathering interest in the research community. Although there are variations internationally, workplace learning typically comprises students learning with, on or about patients/clients; often in the presence of an educator who is a healthcare professional. A few studies have examined constructs and experiences of dignity in healthcare workplace learning (15, 30, 46–49).

For example, investigating students’ and supervisors’ conceptualizations of dignity in workplace learning across healthcare and non-healthcare disciplines (business, counselling, law, teaching, medicine, nursing) research has identified 23 distinct concepts through which participants defined dignity (47), with students being more likely than supervisors to express their conceptualizations in negative terms. Examining participants’ workplace learning experiences, researchers identified nine narrated dignity types: verbal abuse, right for learning opportunities, care, inclusion, reasonable expectations, right for appropriate feedback, equality, trust, and right to be informed (46). Most of these dignity narratives centered around the student-supervisor relationship, fewer on environmental factors, with mainly individual characteristics being cited as contributing factors of dignity experiences (positive and negative). Frequently mentioned supervisor characteristics included expectations of students and feedback competence; frequently mentioned student characteristics included showing initiative, enthusiasm and confidence.

Similarly, Sholl et al. (50) investigated understandings and experiences of hospital clinicians, medical educators and students and public representatives (including simulated patients and lay representatives) about the concepts of safety and dignity in healthcare workplace learning. They identified three types of dignity conceptualizations that interplay with their conceptualizations of safety: physical dignity, emotional and psychosocial dignity, and *other* types of dignity. All of these, except *other* types of dignity, related to respect of self or from others. Differences in understandings between stakeholder groups were not identified.

### Dignity breaches during workplace learning

Research internationally has shed light onto a range of situations in which dignity in healthcare workplace learning is often compromised, including work undertaken within the constructs of *professionalism lapses, professionalism dilemmas* and interactional audio/video research on bedside teaching encounters (26, 39, 40, 42, 44, 45, 50–60). These situations include workplace bullying and harassment, talking to or about patients inappropriately, deliberately withholding information and students conducting examinations (sometimes intimate) on patients without valid consent. Indeed, this classification of professionalism lapses/dilemmas dovetails with the taxonomy of dignity violations identified by Mann (14): not being seen (including being ignored), being reduced to a member of a group category (rather than treated as an individual), violations of personal space, and humiliation. Such compromising of dignity within healthcare workplace learning sometimes happens knowingly, sometimes under duress, and sometimes due to a lack of understanding in how to protect or uphold the dignity of self and others (26, 56).

Workplace learning dignity breaches involve people from different social and professional groups (16, 61) often in unequal relationships with each other where one person is in a dependent, hence vulnerable, position with the other being in a position of power (13, 27, 34, 40, 42, 46, 52, 53). Indeed, unequal relationships exist between a range of groups interacting within the healthcare learning or ‘placement’ environment, including placement educators and students; healthcare professionals and patients; and university staff (who assign, educate, and monitor placement activities) and students. Thus, patients depend on the professionalism of clinicians; with students depending on the guidance, support and assessment of placement educators and university staff.

In addition, students expose their vulnerabilities in the learning process itself. This involves making mistakes as they engage in unfamiliar healthcare workplaces, often for the first time in their lives. In such an environment, dignity breaches can flourish (26); often going unnoticed by those not immediately affected (62, 63), and notoriously being underreported due to feelings of guilt, shame or fear of negative consequences on the side of the individuals whose dignity has been violated (64). As such, dignity breaches can breed further dignity breaches and, over time, erode organizational values and workplace culture, becoming ‘normalized’ or ‘accepted’ ways of behaving (26, 52, 65).

### Consequences of dignity breaches during healthcare workplace learning

In addition to becoming a normalized part of a workplace culture, dignity breaches, especially if continued over time, can have disastrous effects for learners, healthcare professionals and patients. In terms of individual impacts, dignity breaches cause physical, mental and emotional harm (36, 40–43, 52, 55). Indeed, participants report experiencing moral and emotional distress, anxiety and depression, substance abuse, insomnia, physical illnesses, and reduced self-confidence (36, 41). Other consequences include withdrawal and avoidance behaviors, including avoiding perpetrators, avoiding seeking help, or failing to report incidences (41, 43, 52, 56, 66, 67).

Dignity breaches also compromise the wider team in which students and trainees learn alongside organizational performance and productivity, bringing secondary impacts on them (62, 63, 66, 68). For example, recipients of breaches report negative impacts on their job satisfaction, their organizational loyalty, and that they emotionally withdraw from work (30, 61–64).

If patients are on the receiving end, dignity breaches can lead to negative treatment outcomes. For example, patients may lose trust and withdraw from treatment when they hear a healthcare student being involved in disrespectful talking to them or another patient. Similarly, students witnessing or actively being involved in patient dignity breaches can also lead to an increased likelihood of students and/or healthcare professionals making mistakes, e.g., incompetent suturing, (26, 39, 40, 42, 43, 45, 46, 57).

In its essence, dignity is not merely about preventing dignity violations but rather about embracing intrinsic positive qualities and actions. For example, seeing others, acknowledging their individuality, respecting their personal space and honoring social norms (17). Moreover, research shows that many dignity violations in the healthcare workplace are subtle, covert and easily covered up (26), and therefore difficult to articulate. Yet, only an articulatable concept can be applied with confidence (31, 69, 70).

### Study aims

Understanding the range of characteristics of dignity and their sociocultural nuances facilitates an awareness of how to uphold dignity for everyone by identifying what behaviors lead to its violations. Despite previous explorations of the concept of dignity within the workplace learning setting, research has primary focused on a small range of healthcare students’ and placement educators’ perspectives, specifically from medicine and nursing (30, 46, 47, 50). These studies do not account for the wider context in which workplace learning occurs. This context includes a wider range of student and placement educator groups from across allied healthcare settings, and the university staff who source, organize and support placements both educationally and organizationally for whom dignity within workplace learning is a central concern. Indeed, when dignity breaches during workplace learning occur, it is this group (i.e., university staff) who may be required to mediate between students and placement educators and/or their managers, ensuring the welfare of students whilst maintaining overall relationships with the host learning organization.

Extending the range of stakeholders within the practice of healthcare workplace learning interactions therefore brings forth a broader consideration of the triadic interaction between students, placement educators and university workplace learning staff. This accounts for a more rounded understanding of the concept of dignity within workplace learning. Our study aims to fill these gaps in the literature by understanding how allied healthcare students, placement educators and university staff conceptualize the construct of dignity in workplace learning. In doing so we ask the following research questions (RQs): (RQ1) What are participants’ conceptualizations of dignity during workplace learning encounters? (RQ2) How do these understandings differ (if at all) between participants’ stakeholder groups?

## Methods

We used a qualitative, narrative approach, with our underlying theoretical perspective being underpinned by social constructionism. Briefly, social constructionism recognizes that knowledge is created and co-created through talk and interaction. In other words, from an epistemological perspective, social constructionism acknowledges that we come to know our world through social interaction, and this is mediated across different contexts [for full details of the associated ontological, epistemological and axiological underpinnings see (71)]. Narrative approaches sit within the social constructionist world view and are exceptionally useful for uncovering detail and nuance of people’s views and experiences (72). Aligned with this perspective, individual and group discussions were conducted with allied health students, placement educators and university staff whereby participants were asked to provide lived experiences to elucidate their responses. Our initial analysis comprised an inductive thematic approach, commensurate with narrative research undertaken within this genre (73). We received ethics approval from the University of Sydney Human Research Ethics Committee (Project ID 2019/841) and conducted our work according to the submitted protocol that included written and verbal informed consent, participants’ anonymity and their right to withdraw at any time.

### Study context

This study was conducted at a single university setting in Australia that offers allied health professional courses. Allied health disciplines included diagnostic radiography, exercise and sports science, exercise physiology, medical imaging science, occupational therapy, physiotherapy, speech pathology, and rehabilitation counselling. Student participants, who had completed at least one placement in an external placement site were drawn from across these disciplines. Placements, also termed professional practice or practice education, are an integral part of allied health courses, providing students with real-life learning opportunities set within authentic work-based settings Placements are mandated by the various health professions’ regulatory authority professional accrediting bodies, with the aim of ensuring that on completion of their degrees, students are indeed work-ready.

The Work-Integrated Learning (WIL) team at the time of the study comprised both academic and professional staff supporting approximately 5,000 placements for students from allied health professional groups annually. Academic staff are responsible for the design and delivery of the subjects or ‘units of study’ that entirely or partially include a placement component. Unit coordination responsibilities include ensuring students’ preparation for placement, academic support for both students and placement educators during placement, and for some disciplines, debriefing activities following placements. The professional staff members focus on the organizational aspects of the placement, for example, student vaccination verification requirements, student placement allocation, and all operationally-focused communication with placement providers and students. Placement educators comprise healthcare professionals (not employed by the university) whose primary priority is the delivery of healthcare services to members of the public. As part of their professional roles/obligations, these individuals also agree to host placements, supporting student learning and assessment of competency in their setting. Placement providers include publicly funded primary, secondary and tertiary health services, private practices, non-government organizations, and increasingly, large private companies providing services across the National Disability Insurance Scheme or vocational rehabilitation services. The providers are situated both within large metropolitan cities as well as throughout regional and rural areas, and are delivered across the community, accross people’s homes, schools and workplaces, as well as in health settings.

### Recruitment procedure

For placement educators, a stratified purposive sampling approach was employed: the population of placement educators who worked with the study context (> 1,200) was stratified into public, non-government and private organizations, across metropolitan, regional and rural locations with the assistance of a university WIL staff member. All university WIL staff were invited to participate via email from the research assistant (CK), within this invitation they were also asked to nominate a sub-set of up to 20 placement educators from across the stratification. Those nominated were emailed an invitation to participate. Thus, placement educator sampling was purposive in that university WIL staff were asked to select currently active placement educators who were known to them to have had positive and not so positive placement experiences. This ensured that only the most informative placement educators were approached for interviewing. Due to the relatively small number of WIL staff, this sub-sample was recruited using a convenience sampling approach (i.e., every WIL staff member who volunteered to participate was interviewed). The student sub-sample were emailed an invitation. This sub-group was selected purposively with the inclusion criteria that they had completed at least one external work placement during their degree.

### Participants

Fifty-one participants across the three stakeholder groups were interviewed: 19 students, 15 placement educators and 17 work-integrated learning staff (Table 1).

**Table 1:** Overview of participants.

**Characteristic**	**Student**	**Placement educator**	**University WIL staff^**	
			**Academic staff**	**Professional staff**
Gender				
Female	17	11	12	5
Male	2	4	0	0
Age				
Range	21–48	26–54	31–61	40–56
Median	34.5	40.0	46	48
Discipline				
Diagnostic radiography	3	0	3	0
Exercise Physiology/Exercise and Sports Sciences	0	2	1	0
Occupational Therapy	3	5	1	0
Physiotherapy	0	4	3	0
Rehabilitation Counselling	0	1	*	0
Speech pathology	13	3	2	1
Cross disciplinary*	0	0	2	4
First language				
English	12	14	11	5
Non-English	7	1	1	0
Domestic student	14			
International student	5			
Totals (*n* = 51)	19	15	12	5

* Participants working across two or more disciplines are only counted in the ‘Cross disciplinary’ column. ^^^ 12 academic, 5 professional staff.

### Procedure

We held 33 interview sessions: 11 group (comprising 2–4 participants) and 22 individual. All groups, except one, comprised homogeneous participants (i.e., one stakeholder group per session). The exception was one focus group comprising four participants, with two placement educators and two work-integrated learning professional staff located rurally. According to participants’ convenience, we held 16 discussions face-to-face, 16 online (via Zoom) and one interview via the telephone.

Group discussions lasted between 70 and 150 min, the individual interviews between 45 and 100 min. Thirty-one discussion sessions were conducted by CK, two by ABe, neither had a prior relationship with any of participants they interviewed. The discussions were semi-structured with an interview guide beginning by asking participants to define the constructs under study (workplace learning quality and dignity) then moving on to enquire about their experiences of those domains. This study focuses on participants’ responses to the opening question: “*What does the concept of dignity within workplace learning mean to you*?.” When participants responded with an “I think…” answer, we often asked them if they could give us an example of a time when they encountered this as an issue (i.e., asking for a narrative to contextualize their opinions). All discussions were digitally audio-recorded and transcribed anonymously.

### Data analysis

We used a team-based 5-step framework approach to determine content and process-related themes (74). Step 1: All authors read a subset of the transcripts (2–3 each, with each included transcript being read by two people). Step 2: We then discussed our ideas regarding themes identified within the data. Step 3: LVM and CK developed the initial framework document for coding with comments from the wider team. Step 4: The data were then coded by CK with feedback from LVM. Data were managed using the qualitative software ATLAS.ti 8. Three students from outside the study cohort joined the team to help coding the data under the supervision of LVM and CK (see Acknowledgement section). Step 5: LVM and CK further collaboratively developed the wider coding framework through individual coding and whole team discussions. This coding process enabled us to further explore and develop themes and concepts, identifying differences and similarities in understandings across and between the three participant groups.

### Team reflexivity

While the team mainly comprised members of the same university faculty, the project officer CK was neutral in this respect. Team members came from a variety of disciplinary backgrounds, including psychology (LVM), hospital management (CK), higher education (ABe), occupational therapy (MP), physiotherapy (AD) and nutrition (GN). Amongst the team we have a range of expertise in qualitative research from those who have 10–18 years’ experience (LVM, CK, ABe, MP, GN) to those who are relatively novice (AD). Furthermore, as a team we kept check on each other’s interpretations, reminding ourselves of our philosophical framework and being mindful not to go beyond the status of the data.

## Results

We present the explicit, narrated definitions that participants gave us in response to our interview question, “What does the concept of dignity in workplace learning mean to you?,” explicitly addressing RQ1: What are participants’ conceptualizations of dignity during workplace learning encounters? We identified eight explicit definitions of *dignity in workplace learning* and present them below in order of the frequency in which they occurred in the data, except for *dignity as self-x* which we describe immediately after *dignity as respect* where it fits conceptually, although it was mentioned less often (See Table 2, which also addresses RQ2: How do these understandings differ (if at all) between participants’ stakeholder groups?).

**Table 2:** Themes identified by participant group.

	Students (S)	Placement educators (PE)	University WIL staff	
			Academics (WA)	Professional staff (WP)
**Dignity as respect**				
Respect of others	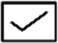	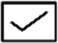	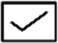	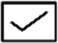
Mutual respect			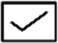	
Culture of respect			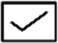	
Dignity as self-x*	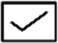	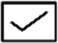	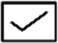	
**Dignity as feeling safe**	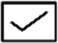	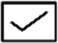	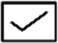	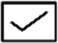
**Dignity as understanding otherness**	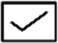	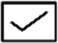	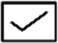	
**Dignity as supporting others**	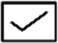	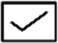	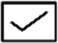	
**Dignity as equality**	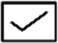	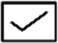	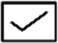	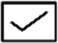
**Dignity as professionalism**	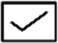	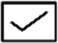	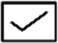	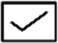
**Dignity as belonging**	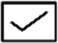	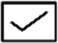	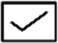	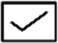

*We use the term “Self-*X*” to refer to the multiple attributes (as commonly denoted by ‘*x*’) of the self this theme comprises.

We order our data pragmatically, making no claim to the relative importance of the concepts (75). Had we interviewed different participants, the frequency of mentions might have been different. When we refer to participants’ talk, we capitalize their participant group name; when a group is referred using lower case, it means that they are the subject of participant’s narrative, e.g., “a Student participant talked of how students on placement….” When we use the phrase “participants talked about *x*,” we mean that an issue was discussed by the *majority* of participants. When we say, “*some* participants narrated *y*,” we are indicating that y was narrated, but not commonly. In the following excerpts, we use a unique identifier denoting participants’ gender (F/M), participant group (Student = ST, Placement Educator = PE, WIL Academic = WA, WIL professional = WP), and participant number.

Before we present the definitions identified, we note that a range of participants from all three stakeholder groups wavered when asked to articulate their conceptualizations of *dignity in workplace learning.* For example, one WIL Academic participant stated that the term “*confuses me a bit*” [F_WA_#6], another WIL Academic participant preferred to use the term *ethical practice* and some Student participants felt that dignity was such an inherent right that it should not need to be defined. Further, participants tended to define dignity from the point of what it is *not*, i.e., by talking about dignity breaches.

### Dignity as respect

Respect was the most common aspect that all participant groups spontaneously associated with the concept of dignity. It was described across multiple dimensions: respect as a characteristic of individuals (respect of others), as a characteristic of interactions (mutual respect) and as a characteristic of the organization/workplace as a whole (culture of respect). It was also described as respect for oneself, but here respect was tightly associated with other qualities such as valuing, understanding and appreciating oneself. We therefore present this aspect under ‘dignity as self-*x*’.

#### Dignity as respect of others

Dignity as respect was most frequently associated with a responsibility of individuals towards others. Respect *towards students* was understood by all participant groups as welcoming students to the site and valuing them as human beings, adult learners and temporary team members rather than judging them in terms of stereotyped demographic characteristics (e.g., perceived nationality based on their name):

One of the negative things I’ve noticed … when you send to sites the names of students [who] are [to be] located to sites, sometimes they [the students] are judged based on their name and there’ve been placements cancelled because the name did not look like the name they expected. That’s not dignity because this student if they’re going there they … would have not been looked at or appreciated the same way they would appreciate others. I did have site cancelling on me placements just after I sent a list of [student] names which were the[type of] names they didn’t expect. [F_WP_#3]

Respect *from students* was understood across multiple dimensions by all participant groups. Firstly, students respecting placement educators’ and team members’ roles and competencies. This includes students knowing their own place and how they fit in within the clinical team. Secondly, students respecting patients’/clients’ needs, including respecting that patients’ bodies are their own, irrespective of students’ need to learn:

That students respect the dignity of their clients or their patients and respect the professionalism of their educators. So I think that can sometimes particularly with graduate entry students be an issue because they may overstep their boundaries as students in terms of learning on placement [F_WP_#2]

Thirdly, in relation to the placement site, students’ respect towards the organisation as a business venture was highlighted.

#### Dignity as mutual respect

Some participants understood respect as *mutual* respect. Here, respect was defined as a relational concept that is exhibited in an interaction, through verbal and non-verbal communication, where both stakeholders take joint responsibility for valuing one another, and treat each other humanely, irrespective of the other person’s status or personal background:

I really do believe [dignity] comes back a lot to communication, and how there is an interaction between the student and the placement educator, or anyone else in the workplace … there has to be a joint accountability and a joint responsibility there with dignity in the workplace. [F_WA_#2]

This understanding came predominately from WIL Academic Staff.

#### Dignity as a culture of respect

Some participants, especially in the WIL Academic Staff group, identified dignity as a *culture of respect* within the workplace. Here, respect was described as being exhibited by *all* members of the organization:

*F_WA_#1: But [respect] has to be- it has to just be there throughout, so a respectful, dignified student going into an environment that —*

*F_WA_#2: --So its almost dignified culture, like the culture already existing there—*

*F_WA_#1: --The culture has to be, is right for the student to go in and be respectful and expect to be respected. [Group Discussion WA_#1]*

### Dignity as self-*x*

This conceptualization was more commonly identified in WIL Academic Staff, Placement Educator and Student participants’ talk and absent in WIL Professional Staff responses. Such an understanding of dignity focuses on the relationship we have with our ‘*self’*: self-worth, self-respect, self-compassion and self-understanding, including an intention to protect one’s sense of self and wellbeing and, in relation to others, an expectation to respect and support this endeavor:

*Believing in yourself, respecting yourself and your position, not feeling like a little junior student out of place, feeling like you have every right to be there, and you belong there and you are doing a great job. That’s the sort of thing that I think would make it dignified if I were a student and I went to a place for learning. [F_CE_#10].*

### Dignity as feeling safe

This understanding of dignity is around physical, mental and emotional safety. All participant groups contributed to this dimension. Students talked about how they had been berated, bluntly being told they are wrong or had done something wrong. Thus, for Students, dignity was about *feeling* safe to learn: to be able to do something without ridicule or being made to feel bad. For Placement Educators and WIL staff it was mostly about *creating* that safe learning space, which was described as one which is free from negative behaviors such as bullying and discrimination, where students can express their wishes and concerns, make mistakes without being penalized and ask “*the dumbest question ever and not be shamed for it*” [F_WA_#8]. So here, the focus is around being mindful of students’ vulnerability due to their status as learners.

Interestingly, one Placement Educator considered the learning process as being inherently unsafe in terms of potentially ‘looking stupid’:

*Well dignity is not something that I would normally attach to a learning process. I think if you maintain- if you, as a student, want to maintain your dignity to a high standard you’re probably not going to learn very well because you’re not prepared to put it out there and give it a go. If I take it away from speech pathology and say learning to do a cartwheel* [a sideways rotary spinning of your body]*, if I’m not prepared to look stupid the first time I do a cartwheel, I am not going to get better [F_PE_#4]*

WIL staff additionally mentioned their own safety and that of placement educators:

People feeling safe and secure and unthreatened and supported in a workplace setting… students and staff… WIL staff yeah, and staff on placement as well, like educators… because we all work together… I see that we work in a triangle [F_WA_#6]

Interestingly, none of the participants linked their explicit understandings of ‘dignity as feeling safe’ to patients.

### Dignity as understanding otherness

Here, all participant groups, except WIL Professional Staff, described dignity in terms of understanding other people’s differences, their individuality, or, in social terms, understanding diversity. This goes beyond respect. It includes giving others space and time to explain their perspective, suspending judgement, and listening deeply to learn from others and to understand them better. Otherness embraces an understanding that people have individual learning needs:

I’m an international student, I come in with different views and different perceptions of what we do in healthcare. So the way that we do stuff might not be the same as compared to what they do over here… Kind of also letting us explain our side of why we perceive it this way, or why we did this, or why we did that [F_ST_#14]

### Dignity as supporting others

Student groups, Placement Educators and WIL Academics highlighted this element. Here, the emphasis is on placement educators supporting their students and giving them opportunities to learn and grow. Several Student and Placement Educator participants spontaneously elaborated on the construct of *dignified feedback* beyond the issue of feeling safe. Placement Educators talked about how they approach feedback in a dignified manner: including making feedback inspiring, empowering, tangible, digestible, keeping it constructive, understanding the intricacies of different students and knowing how to deliver feedback so that it fits individual learners’ needs:

Dignity is about, for students, being able to deliver feedback that’s tailored to the situation and the person when you see how they're reacting. Not just giving them feedback that’s meaningless. Making sure that you show them what you mean. I always jump in sessions and do stuff with them, not berating them but just giving sensible and specific feedback, no personal comments. Giving them a chance to fix it and rewarding anything that they do fix [F_PE_#4]

Dignity as supporting others also includes students’ abilities to accept constructive feedback. It also relates to providing students access to tools, resources and structures that facilitate their learning and undertaking work:

If you don’t give students everything that they need then you’re not giving them the dignity and respect that they deserve and nor are you giving the clients the outcome who you are actually providing services to … students I think can sometimes, if the right structures aren’t in place and the right resources aren’t there, then they can easily be not shown the dignity that they deserve to be able to perform and learn. [F_PE_#11]

This dignity dimension builds on dignity as respect and dignity as understanding otherness in that respect and understanding are now applied practically in deliberate supportive action. Dignity as supporting others also goes beyond merely adhering to professional standards and practice (see *dignity as professionalism*).

### Dignity as equality

The dimension of dignity as equality was highly prevalent in Student participants’ talk, but also common in Placement Educators participants’ talk. It was not mentioned by WIL Professional Staff participants. In this conceptualization, dignity in workplace learning is understood as treating everyone with the *same* level of attention and respect, irrespective of disciplinary or professional hierarchies. Here, the concept of hierarchies and power is often cited by participants, with multiple references across our data to people who are the ‘highest’ and the ‘lowest’ treating each other, and also of being treated as ‘equal.’ Such equality is around the recognition that everyone has a place and relevance in the workspace and is deserving of being respected in that space:

[Dignity] means treatment of their colleagues, respectful communication both verbal, non-verbal, a little bit of a historical thing would be a breakdown of the hierarchy in terms of disciplines or professions. So an EN [Enrolled Nurse] working on the floor with no high level qualifications or someone who is working as a cleaner and/or a ward orderly is given the same level of respect as the highest doctor in your medical team. [M_PE_#2]

In terms of the educator–student relationship this means placement educators treating students as human beings like themselves, and affording all students with the same attention and judgement irrespective of personal preferences, and in turn being treated respectfully by students:

Though everyone has different roles in the scenario, everyone is equal, and equally valued as human… it doesn’t mean the student should not submit to authority when it’s appropriate to do so… they’re learning, and they should be respected as a person that is learning and not ridiculed. [F_WA_#10]

### Dignity as professionalism

This dimension was identified in all participant groups’ talk. Here, dignity was defined as professional behavior using terms such as *being in a privileged position*, *ethics, standards*, *code of conduct, confidentiality, responsibilities*, and *accountability*:

Intrinsically you are dealing with difficulties and so being mindful and respectful of how you approach these subjects so that’s partly about confidentiality, for example, of the information that’s exchanged… that information or anything that’s in that relationship doesn’t flow outside but also that’s additional to all the interpersonal and human respect elements. So that confidentiality applies in organization levels and that recognition that you’re… working from a service provider perspective as being in a privileged position of having access to that information. [F_WA_#5]

Participants also talked about clinicians’ responsibility to communicate professionally, with placement educators and their teams role-modeling professional communication so that students can observe and emulate it. This includes aspects such as having discussions about ethics, patient dignity and putting patients/clients first to ensure the best possible outcomes for them:

Then in a hospital it’s really important to discuss- a lot of our clients are in very difficult stages of life, and it can be hard, and it can sometimes be good to have a discussion about ethics, dignity, how we go about treating these people who really need our help. [F_ST_#4]

I think it’s really around dignity for the client. I think particularly in a workplace in a disability space that we’re putting the client first and really thinking about the supports around them. So I guess really instilling that in the students that come on placement is to see how they are doing in terms of their clinical approach or their therapeutic goal and outcome, how that’s impacting on the person's overall functioning and their life. [F_PE_#9]

### Dignity as belonging

This conceptualization (being identified across all participants groups) also expands on the concepts of ‘dignity as respect’ and ‘dignity as understanding otherness’ in that it focuses on actively including students in the placement team, involving them in activities and providing them with a sense of belonging:

I think inclusiveness, as well, in the team… actually, it felt like being part of the team and they’re not just an add-on for their department or their ward, and they're actually involved in all the activities. That’s around that direct patient contact or clinical skill that they're here to do and that they’re actually involved and valued in that wider team environment as well. [F_PE_#3]

Participants noted that belonging also brings with it a responsibility on the part of the student to work out how to fit in with the site’s team culture, finding a balance between exhibiting professionalism and socializing in a team. In this way, this aspect of dignity also relates to the understanding of ‘dignity as valuing oneself’:

especially within a team environment, knowing how to just be part of that workplace culture around, how to act and have some fun moments, but at the same time be fairly professional, and all of those things and also finding that right balance… So how we manage that as a workplace, but also how the student manages that within themselves. [F_PE_#9]

## Discussion

We identified eight distinct but interrelated dimensions of dignity: dignity as respect, dignity as self-x, dignity as feeling safe, dignity as understanding otherness, dignity as supporting others, dignity as equality, dignity as professionalism, and dignity as belonging. These dimensions we identified in participants’ narratives, combined with existing research around this topic, are invaluable pointers towards what a definition of the concept should entail. We therefore now delineate how our findings compare with existing research, drawing on both the theoretical (philosophical) and the empirical literature with a focus on dignity at work, dignity in healthcare and dignity in workplace learning where appropriate. We offer explanations on how the dimensions of dignity relate to each other; and we discuss implications for future research and practice.

In our data, narratives around *dignity as respect* (with its three sub-dimensions *respect of others*, *mutual respect* and *culture of respect*) and *dignity as self-x* featured prominently. Indeed, the phenomenon of associating dignity with *respect for others* relates to work examining dignity in students and supervisors across healthcare and non-healthcare workplace learning settings (47). However, our analysis takes this further, distinguishing between multiple inward and outward looking aspects of dignity. Such a distinction resonates with that found in the wider conceptual literature across nursing practice. Thus, Gallagher (76) distinguished between ‘dignity as an other-regarding value’ and ‘dignity as a self-regarding value’, referring to mutual respect for others’ and one’s own personal and professional dignity. Jacobson (28) talks about ‘dignity-of-self’ and ‘dignity-in-relation’, the former consisting of self-respect held by individuals but *created through* social interaction; the latter being upheld through a process of reflecting human worth *back* to others through words and actions. Scholars agree on a dialectic relationship between the two perspectives, an internal one (‘how I see [or treat] myself’) and an external one (‘how others see [or treat] me’); both being interdependent, feeding from and into the other through fluid social interaction (34, 77). Our data reflect and extend this interdependent notion to the context of healthcare workplace learning. Thus, within this relational construct dignity arises through one’s own self-respect and treatment of others, which in turn affords others’ recognition of your self-worth and the expectation that you will be treated with similar dignity.

The dimensions of *dignity as feeling safe, dignity as understanding otherness* and *dignity as supporting others* therefore grow out of this interdependent self-otherness, extending beyond merely valuing and respecting others. They require *giving* things that flow from the role one assumes in the workplace: care, understanding and support, including during feedback. Thus, within the workplace learning space the delivering of feedback, often in front of patients/clients, can easily become a context in which patient, student and even educator dignity is compromised with students sometimes left feeling particularly unsafe and vulnerable (56–58). Understanding the nuances around delivering feedback to students in front of patients or team members is key for placement educators’ ability to maintain dignity for all.

So far we have focused on a person-centered, egalitarian perspective of dignity. However, our participants also talked about how different competency and hierarchy levels might call for different ways of behaving, levels of care, understanding and supporting of others. This is where *dignity as professionalism* comes in: described by our participants using attributes like confidentiality, responsibilities, role-modelling, communicating professionally, being in a privileged position, code of conduct, and standards. Indeed, the wider literature describes a similar concept, referring to it as *professional dignity* (16, 32, 78, 79). Thus, professional dignity embraces values such as accountability, excellence, duty, honor, and social identity (16). Professional dignity can be seen as *an achievement* (32) and even as “the sense of pride and accomplishment” associated with one’s profession [(79), p. 41]. Professional dignity has also been associated with rank and status (28, 80) such as *dignity of merit* and *dignity of office* (27, 81, 82). As such, this construct relates to an Aristotelian virtue perspective where through our actions we become honorable and deserve dignity (76).

So, in both, our data and the literature, we find elements that relate to privilege (status, accomplishment, excellence, merit, honor) and elements that relate to responsibility (role-modeling, accountability, communicating professionally). Both sides of *dignity as professionalism*, suggest that one can (or must) *earn* a certain type of dignity at work. This further suggests that some have it and others do not, and that some can claim it and others cannot, depending on professional differences and hierarchies. This conditionality of dignity on instrumental differences between roles makes dignity in workplaces (and for that matter in workplace learning) vulnerable to being misused in that people with more privilege (higher status, competencies, achievements) can take advantage of the dignity of people with less privilege with reference to their privileges/status (15, 34). This usually happens in subtle and tacit ways. By the receiving person it is typically perceived as being looked down upon, being ignored or socially excluded, or by being restricted access to learning opportunities, feedback and resources (*dignity as supporting others*) (34, 37, 41, 42, 46, 47).

It could therefore be argued that a conception of *dignity as professionalism*, as being based on appreciating *differences* between self and others, clashes with the notion of *dignity as equality.* The latter postulates that everybody is of equal worth and that we ought to treat everyone with the same dignity regardless of achievement, skills, status or personal demographics (28). Striking a balance between *dignity as professionalism* and *dignity as equality* is therefore complex, requiring negotiation. As one of our participants commented, while everyone is equally valued, sometimes students do need to “*submit to authority.”* This also explains why our student participants mostly described their conceptualizations of dignity in work-integrated learning with terms that refer to *feeling safe*, *understanding otherness*, *supporting others*, *equality* and *belonging.* Indeed, these dignity dimensions are related predominantly to their relationships with placement educators and clinical team members: all people of higher privilege and status than themselves.

### Methodological limitations and strengths

As with all research, our study has methodological limitations and strengths. Our data was collected using a range of sampling techniques, and we did not scrutinize participants’ motivations to participate in the research before interviewing them. Thus, there are likely to be a range of motivations leading stakeholders to participate, motivations that are likely to be reflected in the data (e.g., after interviewing we note that some participants felt their dignity was breached during workplace learning and were motivated to address the issues through participation). However, this can also be considered as a strength, in that our participants include those with a lived experience of their dignity being compromised and are likely to be an informed sample. A second limitation is that data were collected with students, academic and professional staff at one institution and across six allied health disciplines. Additionally, participants were predominately female (note, there were no male University WIL staff at the time of the study). This limits our findings in terms of generalizability regarding healthcare profession and gender, and therefore our claims. However, the university is the largest in [Australian state] and all clinical educators came from a range of public and private healthcare providers. Furthermore, we recruited a large number of participants (*n* = 51) with a diverse range of workplace learning experiences, and for the first time, included the voices of university academic and professional staff. This range of new voices adds further depth to our findings. Although, we did not include patients’/clients’ perspective into the study, some of our findings touch on dignity in healthcare providers’ and students’ relationships with them. Had we included the patient voice, we might have gleaned an understanding of how they perceive students’ involvement in their care, potentially gaining further insights into how dignified care might be improved. To establish a more comprehensive picture of quality and dignity in work integrated learning, future investigations should include patient/client perspectives (53, 57, 58). The added benefit of an independent qualitative researcher (CK) outside of the university structure (who conducted 29 of the 31 interview sessions), meant that participants were able to discuss their experiences candidly and in complete anonymity. Finally, our team was able to work with several students (Pharmacy and Public Health) who brought fresh eyes and a student perspective to the study (see Acknowledgements).

#### 4.1.1 Educational recommendations

Our research offers a number of suggestions for the promotion of dignity within workplace learning settings. Firstly, given that participants’ understandings are wide-ranging and complex, diverging across workplace learning stakeholder groups, we suggest reviewing existing dignity concepts underpinning healthcare workplace learning curricula with a focus on ensuring three characteristics: (1) that it is communicated in a way that emphasizes positive actions, enabling stakeholders to understand, recognize and uphold dignity in workplace learning; (2) that it enables students, educators and sites to recognize, report and manage dignity violations; and (3) that it explicitly addresses any tension between dignity as professionalism and as equality, offering solutions for all stakeholders to navigate this tension skillfully and appropriately. Importantly, understanding the interdependent nature of self and other dignity is key to this tension. This includes reinforcing with educators that making students feel welcome and safe, integrating them into workplace teams, accommodating to their capabilities, supporting them in their learning and granting them access to learning opportunities and resources, and constructive feedback are essential elements of dignity in workplace learning (46, 47) and duties arising from professionalism at work. Indeed, in terms of feedback, video ethnographic research examining interactional intricacies of feedback sequences has identified a range of strategies that serve to exclude the learner [e.g., overtly direct, very discrete or out of context: 84] whereby students might consider that their learning is being ignored [i.e., the indignity of not being seen: 14]. This research however also identifies a number of interactionally and educationally effective *embedded* strategies for the provision of timely feedback (83). These strategies uphold dignity through face-saving activities within the triadic clinician-student-patient/client encounter by fostering positive student participation, sensitively correcting and minimizing students’ errors and developing self-esteem within specifically tailored learning opportunities. Indeed, it has been argued that “unequal power relations can be minimized and an aura of joint ‘learning’ experiences can be facilitated” though the use of embedded feedback with sensitive correction strategies [(83), p. 519].

We extend the promotion of dignity within workplace learning settings to the co-construction of student learning experiences that benefit all stakeholders: patients, students, service and education providers. Co-designing from a service delivery perspective, with an emphasis on how students can add value to the organization *through* participation (84) has potential to positively impact workplace dignity. In other words, students become a useful resource and in turn feel useful.

While there are no easy solutions, we further suggest providing all workplace learning stakeholders with suitable learning opportunities where they can practice applying the multi-dimensional dignity concept, collaboratively developing and strengthening a culture of dignity in workplace learning across healthcare settings. Through the development of this positive dignity culture, breaches can be reduced across healthcare workplace learning spaces, enabling students, educators and university staff to be equipped with more effective tools to embrace and live a dignified workplace learning culture.

## Data availability statement

The datasets presented in this article are not readily available because ethics approval was contingent on data being stored on a secure university repository. We did not obtain permission for data sharing.

## Ethics statement

The studies involving humans were approved by The University of Sydney Human Research Ethics Committee (Project ID 2019/841). The studies were conducted in accordance with the local legislation and institutional requirements. The participants provided their written informed consent to participate in this study.

## Author contributions

CK: Formal analysis, Investigation, Software, Writing – original draft, Writing – review & editing. AD: Formal Analysis, Investigation, Writing – original draft, Writing – review & editing. AB: Formal analysis, Investigation, Writing – original draft, Writing – review & editing. GN: Formal analysis, Investigation, Writing – original draft, Writing – review & editing. MP: Formal analysis, Investigation, Writing – original draft, Writing – review & editing. LM: Conceptualization, Formal analysis, Funding acquisition, Investigation, Methodology, Supervision, Writing – original draft, Writing – review & editing.
